# Unmasking yamaguchi syndrome: A rare case of apical hypertrophic cardiomyopathy in a young African - American male

**DOI:** 10.6026/973206300210257

**Published:** 2025-02-28

**Authors:** Sharanya Rajgopa, Yashkumar Chauhan, Keerthi Talluri, Safa Kaleem, Salman Sajid, Tejashwini Reddy, Ankur Shah

**Affiliations:** 1 Department of Internal Medicine, NYU Langone Health Hospital, New York, United States of America

**Keywords:** Yamaguchi syndrome, cardiomyopathy, cardiovascular magnetic resonance, left ventricular hypertrophy, diagnostic imaging

## Abstract

Yamaguchi syndrome, also known as apical hypertrophic cardiomyopathy (AHCM), is a genetic disorder predominantly affecting the apex of the left ventricle and often presenting similarly to acute coronary syndrome, making precise imaging crucial for diagnosis. This condition, first identified in Japanese populations, is more common in Asian communities but varies in frequency across different populations. We are presenting the case of a 30-year-old African-American male patient with a history of hyper-lipidemia, asthma and obesity, who reported palpitations, dizziness and chest pain radiating to the left arm and jaw, particularly under stress. Echocardiography and cardiovascular magnetic resonance (CMR) revealed severe left ventricular hypertrophy, mild valvular regurgitation and marked apical obliteration, confirming the diagnosis of apical hypertrophic cardiomyopathy. This case highlights the need to consider apical hypertrophic cardiomyopathy in the differential diagnosis of patients with hypertrophic features, especially when conventional imaging findings are unclear.

## Background:

Yamaguchi syndrome, another name for apical hypertrophic cardiomyopathy, was initially identified in Japanese people in the 1970s [1]. It is a rare genetic illness that affects the apex and distal regions of the left ventricle. It is distinguished by asymmetric involvement of the left ventricle and non-obstructive hypertrophic cardiomyopathy [2]. Cardiac damage, including cardiac myocyte disarray and extracellular matrix alterations, is the primary mechanism responsible for myocardial fibrosis. Hypertrophic cardiomyopathy is caused by mutations in sarcomere proteins, namely myosin-binding protein C (MYBPC3) and β-myosin heavy chain (MYH7) [3]. Apical hypertrophic cardiomyopathy varies in frequency among populations; in Asian communities, it is around 40% more common than in North American and European populations (8%) [4]. It's distribution is also more common in men than in women, with an average age of presentation of 41.4 ± 14.5 years [5]. As demonstrated in this instance, its clinical manifestation can resemble acute coronary syndrome, highlighting the significance of imaging in the precise identification of apical hypertrophic cardiomyopathy [1]. The diagnosis of apical hypertrophic cardiomyopathy is made using echocardiography results, which have limitations in identifying this illness. Because of its exceptional diagnostic precision and efficacy in detecting and evaluating apical hypertrophic cardiomyopathy, cardiac magnetic resonance imaging is considered the gold standard [6]. In this case report, we present a young African-American male diagnosed with apical hypertrophic cardiomyopathy, highlighting clinical and diagnostic challenges.

## Case description:

**Figure 1:**
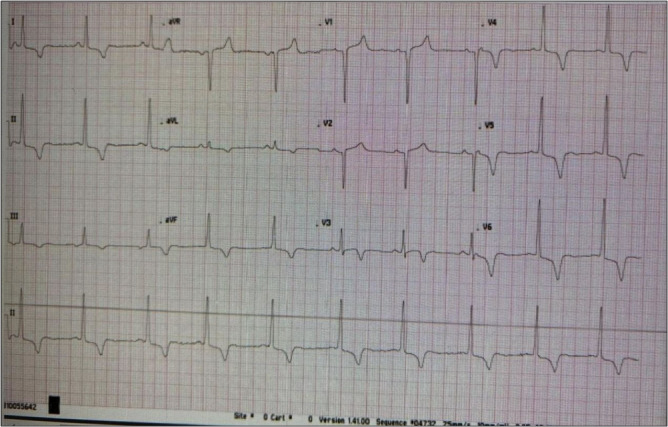
EKG showing changes related to hypertrophic cardiomyopathy

An African-American male patient, aged 30, with a medical history of hyperlipidemia, asthma and obesity, visited the clinic with complaints of palpitations and dizziness occurring once every 1-2 weeks. He abstains from smoking and consuming alcohol. He abstains from using any recreational substances. He has not undergone any surgical interventions and there is no familial history of sudden cardiac death. The patient is administering albuterol sulfate 1.25 mg/3ml inhalation nebulization solution. The patient has asthma and is taking a 20 mg oral pill of atorvastatin calcium for hyperlipidemia. Additionally, he is taking a daily dose of 50 mg of losartan. The prescribed medication for hypertension is hydrochlorothiazide, taken once daily at a dosage of 25 mg. additionally, diltiazem HCl is prescribed in the form of an oral tablet with a strength of 420 mg. The medication prescribed is metoprolol succinate ER 50 mg oral tablet, which is an extended-release formulation designed to have a 24-hour effect. It is used to treat hypertrophic cardiomyopathy. During the clinic visit, the patient's physical examination indicated a high blood pressure reading of 182/96 mmHg, a pulse rate of 70 beats per minute and a body mass index of 35.05. The patient's review of systems; the central nervous system, respiratory system, gastrointestinal system, musculoskeletal system, as well as the genitourinary system, showed no abnormalities. The report of the patient's color Doppler echocardiography is showing mild aortic regurgitation. There is a mild condition of mitral regurgitation. There is an abnormality in the diastolic function. There is a mild backflow of blood via the tricuspid valve. The left ventricle is enlarged, but its function is normal. The left atrium is enlarged, while the right atrium is of regular size disordered diastolic function. The patient has significant left ventricular hypertrophy (LVH) with modest systolic anterior motion (SAM) and left ventricular outflow tract (LVOT) obstruction, with a mean pressure gradient of 4 mmHg. The report of the patient's exercise echocardiography is as follows: The patient had a moderate exercise capacity and a typical heart rate and blood pressure response. EKG response to exercise without ischemia and normal heart rate recovery and there is no indication of any major blockage in the coronary arteries (Figure 1). The ECG shows sinus rhythm with T wave inversion (Table 1).

## Discussion:

A rare kind of hypertrophic cardiomyopathy (HCM), apical hypertrophic cardiomyopathy (AHCM) often affects the left ventricular apex and seldom the right ventricular apex, or both [7]. Apical hypertrophic cardiomyopathy is mostly sporadic; however few cases with autosomal dominant inheritance have also been documented [8]. Three subtypes of apical hypertrophic cardiomyopathy exist morphologically: pure focal, pure diffuse and mixed [9]. It is unclear if this sub classification has any clinical significance and is not generally recognized in clinical practice. Others have divided apical hypertrophic cardiomyopathy into two categories according to whether they had coexisting interventricular septal hypertrophy (mixed AHCM) or isolated asymmetric apical hypertrophy (pure AHCM) [10]. The most frequent presenting symptom of apical hypertrophic cardiomyopathy is chest pain, followed by palpitations, dyspnea and syncope. The mean age of presentation is 41.4 ± 14.5 years and it is more usually observed in males [11].

Other symptoms of apical hypertrophic cardiomyopathy include cardiac arrest, apical aneurysm, ventricular fibrillation and congestive heart failure, myocardial infarction, embolic events and atrial fibrillation [10]. This case is about a 30-year-old African American male with hyperlipidemia, asthma and morbid obesity who presents with symptoms of chest pain, dizziness and palpitations. Initially, he had episodes of palpitations associated with dizziness once every 1-2 weeks for the past few months. He experienced new-onset left-sided sharp chest pain (6-10) radiating to the left arm and jaw associated with palpitations, dizziness and shortness of breath, which worsened with exertion and stress but was relieved with aspirin. This case demonstrates the presentation of apical hypertrophic cardiomyopathy mimicking CAD, highlighting the clinical and diagnostic challenges. Over time, the diagnostic standards for apical hypertrophic cardiomyopathy have changed. Initially, the diagnosis required left ventriculography exhibiting a "unique spade-like configuration and marked apical obliteration" in addition to electrocardiographic "giant" negative T-waves and high QRS voltage. [11]. With advancements in imaging, the criteria for diagnosis currently consist of LVH predominating in the LV apex, with wall thickness in the apex ≥15 mm and a maximum apical to posterior wall thickness ratio ≥1.5, on echocardiography or cardiovascular magnetic resonance (CMR) [10]. The initial test of choice for diagnosing is transthoracic echocardiography, which can detect apical hypertrophy, distinguish between pure and mixed forms and identify other prognostic factors that may influence the outcome, such as diastolic dysfunction, mid ventricular obstruction with cavity obliteration (MVOCO), or apical aneurysms [12, 13]. Cardiovascular magnetic resonance (CMR) is more sensitive in identifying apical aneurysms in individuals with ECG abnormalities but inconclusive echocardiography. The EKG of our patient showed sinus rhythm with T-wave inversions. Echocardiography revealed a dilated left ventricle and left atrium with abnormal diastolic function. LVH is severe, revealing hypertrophic cardiomyopathy, with mild SAM and an LVOT PG mean of 4 mmHg. The TMT revealed no echocardiographic findings of significant CAD. Cardiovascular magnetic resonance revealed LVH predominantly in mid- to apical segments and hyper-dynamic LV with nearly complete cavity obliteration of apical LV during systole, thereby aiding in diagnosing apical hypertrophic cardiomyopathy. This case demonstrates the challenges faced in diagnosing apical hypertrophic cardiomyopathy, its presentation similar to that of CAD and the sensitivity of cardiovascular magnetic resonance in diagnosing apical hypertrophic cardiomyopathy. The presentation of apical hypertrophic cardiomyopathy can be asymptomatic, an incidental finding, or present as CAD. So a high degree of suspicion should be established when managing patients with clinical symptoms and hypertrophy on echocardiography [14]. Apical cardiomyopathy, a subtype of hypertrophic cardiomyopathy, necessitates a comprehensive management strategy that encompasses lifestyle adjustments, medication and sometimes invasive interventions. Patients are encouraged to avoid strenuous physical activities and extreme stress to prevent symptom exacerbations and reduce the risk of complications [15]. Regular follow-up using echocardiography and other imaging techniques are crucial to assessing disease progression and the efficacy of the treatment regimen [16]. Pharmacological treatment is a key component of managing apical cardiomyopathy. Beta-blockers and calcium channel blockers are typically prescribed to alleviate symptoms such as chest pain and palpitations by reducing myocardial oxygen demand and controlling heart rate [16]. Anticoagulants are recommended if there is a risk of thromboembolism, particularly in patients with atrial fibrillation [17]. Additionally, antiarrhythmic drugs are utilized to manage significant and potentially life-threatening arrhythmias [18]. In some cases, invasive procedures may be necessary. Septal reduction therapy, including septal myectomy or alcohol septal ablation, is considered for patients with substantial outflow tract obstruction and severe symptoms that are unresponsive to medication [19]. Implantable cardioverter-defibrillator (ICD) implantation is advised for patients at high risk of sudden cardiac death [20, 21].

It can be summarized that Hypertrophic cardiomyopathy is an autosomal dominant condition affecting the heart muscle, mainly due to mutations in sarcomeric protein genes [22]. A notable variant is apical hypertrophic cardiomyopathy, also known as Yamaguchi syndrome, which primarily impacts the apex of the left ventricle [23]. ApHCM is characterized by distinct electrocardiographic findings like "giant" negative precordial T-waves and a "spade-like" left ventricle configuration seen in imaging studies. Diagnosis can be challenging due to its rarity and similarity to other conditions, requiring genetic testing for confirmation [24, 25]. Management focuses on relieving symptoms, preventing complications, and tailoring treatment plans based on disease severity. ApHCM carries a risk of arrhythmias and sudden cardiac death, emphasizing the importance of early detection and intervention for better patient outcomes.

## Conclusion:

Apical hypertrophic cardiomyopathy is an often under-recognized subtype of hypertrophic cardiomyopathy that can resemble coronary artery disease, requiring careful differentiation. Advances in cardiovascular magnetic resonance imaging enhance apical hypertrophic cardiomyopathy diagnosis by revealing features like apical obliteration. Effective management involves tailored lifestyle modifications, pharmacological treatments, potential interventions and regular monitoring through echocardiography and cardiovascular magnetic resonance.

## Figures and Tables

**Table 1:** Medical timeline

**Date**	**Event/Diagnosis**	**Details**
Several months ago	Symptom Onset	Patient began experiencing palpitations and dizziness once every 1-2 weeks, with heart racing, fluttering and episodes of dizziness, blurry vision and blackouts.
2 weeks ago	New Symptom Onset	Patient started experiencing intermittent, pressure-like chest pain on the left side, radiating to the left arm and jaw, rated 6/10 in intensity, worse with stress and physical activity, relieved by sitting and aspirin. Also reports shortness of breath with chest pain, especially when walking.
Today	Clinic Visit	- Vitals: BP 182/96 mmHg, pulse 70 bpm, BMI 35.05. - Review of Systems: Normal for CNS, respiratory, gastrointestinal, musculoskeletal, genitourinary.
Today	Diagnostic Test: Color Doppler Echocardiogram	- Trivial aortic regurgitation - Mild mitral regurgitation - Mild tricuspid regurgitation - Dilated left ventricle with normal function - Normal right ventricle size and function - Dilated left atrium; normal right atrium size - Abnormal diastolic function - Severe LVH - Hypertrophic cardiomyopathy with mild SAM and LVOT pressure gradient mean 4 mmHg
Today	Diagnostic Test: Exercise Echocardiogram	- Average exercise tolerance - Normal chronotropic and BP response - Non-ischemic EKG response to exercise - Normal heart rate recovery - No echocardiographic evidence of significant coronary artery disease - ECG shows sinus rhythm with T wave inversion
Today	Diagnosis: Hypertrophic Cardiomyopathy	Severe LVH, mild SAM and mild LVOT pressure gradient noted on echocardiogram.
Today	Diagnosis: Uncontrolled Hypertension	BP recorded at 182/96 mmHg.
Today	Diagnosis: Hyperlipidemia	Patient is on atorvastatin 20 mg daily.
Today	Diagnosis: Obesity	BMI recorded at 35.05.
Today	Diagnosis: Asthma	Patient uses albuterol sulfate as needed.
Today	Diagnosis: New Onset Chest Pain	Intermittent, pressure-like chest pain, radiating to left arm and jaw, associated with shortness of breath, worse with stress and physical activity.
Today	Plan: Hypertrophic Cardiomyopathy	Continue current medications, advise symptom log, schedule regular follow-ups, consider referral to a cardiologist.
Today	Plan: Hypertension	Adjust antihypertensive therapy, encourage home BP monitoring, discuss lifestyle modifications.
Today	Plan: Hyperlipidemia	Continue atorvastatin 20 mg daily, check lipid profile at next visit.
Today	Plan: Obesity	Recommend weight management program, track weight and BMI at each visit.
Today	Plan: Asthma	Continue albuterol sulfate as needed, assess and adjust treatment if necessary.
Today	Plan: New Onset Chest Pain	Perform ECG and check cardiac enzymes, consider further testing like coronary CT angiography if symptoms persist.
Today	Patient Education and Additional Considerations	Educate about medication adherence, lifestyle changes, recognizing symptoms requiring immediate medical attention, consider mental health screening for anxiety/stress.
